# Vasoplegia after implantation of a continuous flow left ventricular assist device: incidence, outcomes and predictors

**DOI:** 10.1186/s12871-018-0645-y

**Published:** 2018-12-08

**Authors:** Eric E. C. de Waal, Bas van Zaane, Marnix M. van der Schoot, Albert Huisman, Faiz Ramjankhan, Wilton A. van Klei, Nandor Marczin

**Affiliations:** 1 Department of Anesthesiology, University Medical Centre Utrecht, Mailstop Q04.2.317, Post Office Box 85500, 3508 GA Utrecht, Netherlands; 2 Medical student, Faculty of Medicine, Utrecht University, Utrecht, The Netherlands; 3 Clinical chemist, Department of Clinical Chemistry and Hematology, University Medical Centre Utrecht, Utrecht, The Netherlands; 4 Cardiothoracic surgeon, Department of Cardiothoracic Surgery, University Medical Centre Utrecht, Utrecht, The Netherlands; 5 Anaesthesiologist, Section of Anaesthesia, Pain Medicine and Intensive Care, Imperial College, London, UK; 6 Department of Anaesthesia and Intensive Care, Semmelweis University, Budapest, Hungary

**Keywords:** Cardiac Vasoplegia syndrome, Mechanical circulatory support, Incidence, Outcome, Morbidity, Mortality, Prediction

## Abstract

**Background:**

Vasoplegia after routine cardiac surgery is associated with severe postoperative complications and increased mortality. It is also prevalent in patients undergoing implantation of pulsatile flow left ventricular assist devices (LVAD). However, less is known regarding vasoplegia after implantation of newer generations of continuous flow LVADs (cfLVAD). We aim to report the incidence, impact on outcome and predictors of vasoplegia in these patients.

**Methods:**

Adult patients scheduled for primary cfLVAD implantation were enrolled into a derivation cohort (*n* = 118, 2006–2013) and a temporal validation cohort (*n* = 73, 2014–2016). Vasoplegia was defined taking into consideration low mean arterial pressure and/or low systemic vascular resistance, preserved cardiac index and high vasopressor support. Vasoplegia was considered after bypass and the first 48 h of ICU stay lasting at least three consecutive hours. This concept of vasoplegia was compared to older definitions reported in the literature in terms of the incidence of postoperative vasoplegia and its association with adverse outcomes. Logistic regression was used to identify independent predictors. Their ability to discriminate patients with vasoplegia was quantified by the area under the receiver operating characteristic curve (AUC).

**Results:**

The incidence of vasoplegia was 33.1% using the unified definition of vasoplegia. Vasoplegia was associated with increased ICU length-of-stay (10.5 [6.9–20.8] vs 6.1 [4.6–10.4] *p* = 0.002), increased ICU-mortality (OR 5.8, 95% CI 1.9–18.2) and one-year-mortality (OR 3.9, 95% CI 1.5–10.2), and a higher incidence of renal failure (OR 4.3, 95% CI 1.8–10.4). Multivariable analysis identified previous cardiothoracic surgery, preoperative dopamine administration, preoperative bilirubin levels and preoperative creatinine clearance as independent preoperative predictors of vasoplegia. The resultant prediction model exhibited a good discriminative ability (AUC 0.80, 95% CI 0.71–0.89, *p* <  0.01). Temporal validation resulted in an AUC of 0.74 (95% CI 0.61–0.87, *p* <  0.01).

**Conclusions:**

In the era of the new generation of cfLVADs, vasoplegia remains a prevalent (33%) and critical condition with worse short-term outcomes and survival. We identified previous cardiothoracic surgery, preoperative treatment with dopamine, preoperative bilirubin levels and preoperative creatinine clearance as independent predictors.

**Electronic supplementary material:**

The online version of this article (10.1186/s12871-018-0645-y) contains supplementary material, which is available to authorized users.

## Background

Vasoplegia is characterized by severe hypotension due to lack in vasomotor tone refractory to catecholamine therapy. It occurs frequently after routine cardiac surgery (up to 27%) [1–6] and is associated with severe postoperative complications and increased mortality. Vasoplegia is even more prevalent after advanced surgical treatment of heart failure. The incidence of vasoplegia after heart transplantation (HTx) ranged between 8.8 and 54%, depending on the definition used [7–9], and 42% after pulsatile left ventricular assist device (pLVAD) implantation [10]. Less is known regarding vasoplegia after insertion of the newer generation of continuous flow LVADs (cfLVAD).

Published definitions for vasoplegia post-cardiac surgery vary markedly and include different hemodynamic parameters, vasoactive drugs, patient groups and variable observed time periods, which may lead to differences in reported incidences [1, 3, 8, 9, 11, 12] and associations with relevant clinical outcomes [1–6, 13]. Careful analysis of these definitions reveals that their application to the cfLVAD population is limited. Better identification of patients suffering from vasoplegia in this population and a better prediction of this critical condition might lead to improved optimization and result in improved outcomes. Therefore, for this specific patient population an appropriate and more unified definition taking into consideration high vasopressor requirements to maintain normal SVR and an extension of the time frame to the first 48 postoperative hours seems required.

As the newer generation of cfLVADs appears to improve postoperative survival [1–9, 14], we hypothesized that their implantation might be associated with a reduced incidence of vasoplegia, contributing to a better clinical course especially in the early postoperative period compared to older generations of LVADs, such as pLVADs.

Based on these considerations, the first aim of the study was to uncover the incidence of vasoplegia using the unified definition and to compare this incidence to incidences obtained with three previously published definitions [1, 2, 9]. In addition, we set out to define the association of vasoplegia with several clinical outcomes including mortality, and to identify independent preoperative predictors of vasoplegia.

## Methods

This study was performed in accordance with the declaration of Helsinki. Prior to data collection the study protocol was assessed by the institutional review board of the University Medical Centre Utrecht, The Netherlands and approved with an exemption from requiring ethical approval (14–053/C) because patients were not subjected to any investigational action.

### Inclusion and exclusion criteria

We included patients > 18 years, suffering from slowly deteriorating chronic or acute (on chronic) heart failure, scheduled for short-term (Centrimag, St. Jude Medical, St. Paul, MN, USA) or long-term (Heartmate II or Heartmate III, St. Jude Medical, St. Paul, MN USA; or Heartware, HeartWare Inc., Framingham, MA, USA) cfLVAD implantation. As the study was focusing on first time cfLVAD implantation, we excluded patients with an already inserted assist device and patients with intraoperative right ventricular (RV) failure requiring a RV assist device implantation (RVAD) during the primary cfLVAD implantation procedure. A derivation cohort of patients operated in the period 2006–2013 was used for development of the prediction model, while this prediction model was temporally validated in a cohort of patients scheduled for cfLVAD implantation in our hospital in the period 2014–2016.

### Data collection

Procedure related data were extracted from our anaesthesia information system (Anstat, Carepoint, Ede, The Netherlands), the electronic hospital information system (EZIS, ChipSoft, Amsterdam, The Netherlands) and the intensive care unit (ICU) data monitoring system (Metavision, iMDsoft, Düsseldorf, Germany).

### Definitions

In order to estimate the incidence of vasoplegia after cfLVAD implantation, we applied previously published definitions of vasoplegia [1–3, 8–11] (Table 1). As these definitions vary significantly in their hemodynamic criteria and included different postoperative time frames, we have attempted to simplify and unify these definitions by highlighting the common basic hemodynamic issue of low SVR and/or MAP in the setting of high vasopressor requirements (as used in the other definitions) with retention of a normal cardiac index (CI), covering the first 48 h after arrival in the ICU. Therefore, we constructed a unified definition and considered patients as vasoplegic if they had following conditions for at least three consecutive hours during the first 48 h after ICU arrival: a vasodilation criterion: MAP ≤50 mmHg or SVR ≤800 dynes·s·cm^− 5^; a hemodynamic criterion: CI ≥ 2.5 l·min^− 1^·m^− 2^; high vasopressor requirement: use of norepinephrine ≥200 ng·kg^− 1^·min^− 1^ or equivalent doses of vasopressors (epinephrine ≥200 ng·kg^− 1^·min^− 1^; dopamine ≥30 μg·kg^− 1^·min^− 1^; phenylephrine ≥2 μg·kg^− 1^·min^− 1^, or vasopressin ≥0.08 U·min^− 1^) as proposed in the ATHOS-3 trial [15].

**Table 1:** Overview of definitions of vasoplegia used and their criteria

	Vasodilation criterion	Hemodynamic criterion	Vasopressor criterion	Preload criterion	Time moment/period
Argenziano [1]	MAP < 70 mmHg	CI > 2.5 l·min^− 1^·m^− 2^	nor > 8 μg·min^− 1^		5 min after CPB
Levin [2]	MAP < 50 mmHg and SVR < 800 dynes·s·cm^− 5^	CI > 2.5 l·min^− 1^·m^− 2^	any vasopressor	CVP < 5 mmHg and PCWP < 10 mmHg	first 3 h after ICU arrival
Patarroyo [9]	SVR < 800 dynes·s·cm^− 5^	CI > 2.5 l·min^− 1^·m^− 2^	≥ 2 vasopressors: 1. epi > 4 μg/min, 2. nor ≥4 μg/min, 3. dopa ≥5 μg· kg^− 1^·min^− 1^, 4. vasopressin ≥1 U/hr		6–48 h after ICU arrival
Unified definition	MAP < 50 mmHg or SVR < 800 dynes·s·cm^− 5^	CI > 2.5 l·min^− 1^·m^− 2^	nor > 200 ng·kg^− 1^·min^− 1^ or equivalent doses of other vasopressors: epi ≥ 200 ng·kg^− 1^·min^− 1^; dopa ≥30 μg·kg^− 1^·min^− 1^; phenyl ≥2 μg·kg^− 1^·min^− 1^; or vasopressin ≥0.08 U·min^− 1^		first 48 h after ICU arrival

Thermal filament Continuous Cardiac output was measured with a pulmonary artery catheter (Type 744F75, Edwards Lifesciences, Irvine, California, USA) and a CI ≥ 2.5 l·min^− 1^·m^− 2^ was used in order to exclude other possible causes of hypotension and “vasoplegia”, such as right ventricular failure and hypovolemia.

To relate vasoplegia to doses of various administered vasoactive drugs, the Vasoactive Inotropic Score (VIS) [16] was calculated before the operation and during every hour in the first 48 postoperative hours: VIS = dopamine dose (μg·kg^− 1^·min^− 1^) + dobutamine dose (μg·kg^− 1^·min^− 1^) + 100 x epinephrine dose (μg·kg^− 1^·min^− 1^) + 10 x milrinone dose (μg·kg^− 1^·min^− 1^) + 10,000 x vasopressin dose (U·kg^− 1^·min^− 1^) + 100 x norepinephrine dose (μg·kg^− 1^·min^− 1^) + 10 x phenylephrine dose (μg·kg^− 1^·min^− 1^) [16]. Maximum VIS scores during the first and second 24 h after ICU arrival were used in the analysis.

### Outcomes

For all used definitions, the primary endpoint was the incidence of vasoplegia after cfLVAD implantation, while secondary endpoints were renal failure [17], stroke [18], gastrointestinal bleeding, pneumonia, delirium and resternotomy for bleeding or tamponade, ICU length of stay (ICU-LOS), ICU-mortality, Post-ICU-Hospital LOS; 30-days and 1-year mortality. Renal failure was defined as an abrupt (within 48 h) reduction in kidney function with an absolute increase in serum creatinine of more than or equal to 0.3 mg/dl (≥26.4 μmol/l), a percentage increase in serum creatinine ≥50% (1.5-fold baseline), or a reduction in urine output (documented oliguria of less than 0.5 ml/kg/hour for more than 6 h).

### Statistical analysis

Statistical analysis was performed using SPSS version 24 for Mac (SPSS Inc., Chicago, IL, USA). Continuous variables are presented as mean ± standard deviation, or median [interquartile range]. Categorical variables are summarized as counts and percentages. All definitions were applied to estimate the incidence of postoperative vasoplegia and its association with outcomes after primary cfLVAD implantation. Univariable and multivariable logistic regression analyses were used to identify independent preoperative predictors of vasoplegia. A Cox-regression survival curve censored at cfLVAD removal for recovery, replacement or HTx was generated to explore a difference between both groups using the unified definition. For the unified vasoplegia definition, we used a cut-off *p*-value of 0.05 for inclusion of potential pre-operative predictors for the development of the final prediction model. Results are presented as odds ratio (OR) with 95% confidence interval (95%CI). The ability of predictors to discriminate patients with postoperative vasoplegia was quantified by calculating the area under the receiver operating characteristic curve of the predictor obtained from the multivariable regression model [19]. In addition, the obtained prediction model was temporally validated in a second cohort of patients operated on in the period 2014–2016 [20, 21].

## Results

Demographic data, indication for cfLVAD implantation and medical history of 118 included patients in the derivation cohort are presented in Table 2.

**Table 2:** Demographic data, indication for cfLVAD-implantation and medical history of patients included in the derivation and the validation cohort. Values are expressed as numbers and % of patients, mean ± SD, or median [Interquartile Range]

	Derivation cohort 118 patients	Validation cohort 72 patients
Demographic data		
Age (years)	49.4 ± 12.8	51.2 ± 13.0
Male gender	83 (70.3%)	49 (68.1%)
Weight (kg)	75.0 ± 14.0	75.6 ± 15.0
Height (cm)	177 ± 9	174 ± 23
BSA (m^2^)	1.92 ± 0.19	1.92 ± 0.22
BMI (kg·m^−2^)	23.9 ± 4.1	24.1 ± 3.8
Indication LVAD implantation		
Hypertrophic CMP	3 (2.5%)	1 (1.4%)
Ischemic CMP	25 (21.2%)	16 (22.2%)
Non-compaction CMP	5 (4.2%)	0 (0.0%)
Dilating CMP	85 (72.0%)	52 (72.2%)
Myocarditis	0 (0.0%)	1 (1.4%)
Congenital CMP	0 (0.0%)	1 (1.4%)
Toxic CMP	0 (0.0%)	1 (1.4%)
Medical History		
Diabetes Mellitus	9 (7.6%)	10 (13.9%)
Hypertension	10 (8.5%)	10 (13.9%)
Hypercholesterolemia	12 (10.2%)	11 (15.3%)
Smoking history	64 (54.2%)	25 (34.7%)
Thyroid disease	15 (12.7%)	7 (9.7%)
COPD	13 (11.0%)	9 (12.5%)
Stroke	7 (5.9%)	4 (5.6%)
Transient Ischemic Attack	6 (5.1%)	3 (4.2%)
Previous Cardiothoracic surgery	14 (11.9%)	13 (18.1%)
HMIIRS	2.4 ± 1.3	1.5 ± 1.0
Euroscore II	22.1 [17.3–34.0]	19.0 [12.3–34.7]
Intermacs Class		
Intermacs class I	15 (12.7%)	1 (1.4%)
Intermacs class II	63 (53.4%)	31 (43.1%)
Intermacs class III	33 (28.0%)	27 (37.5%)
Intermacs class IV	7 (5.9%)	12 (16.7%)
Intermacs class V	0 (0.0%)	0 (0.0%)
Intermacs class VI	0 (0.0%)	1 (1.4%)

### Incidence of vasoplegia

**Fig. 1:**
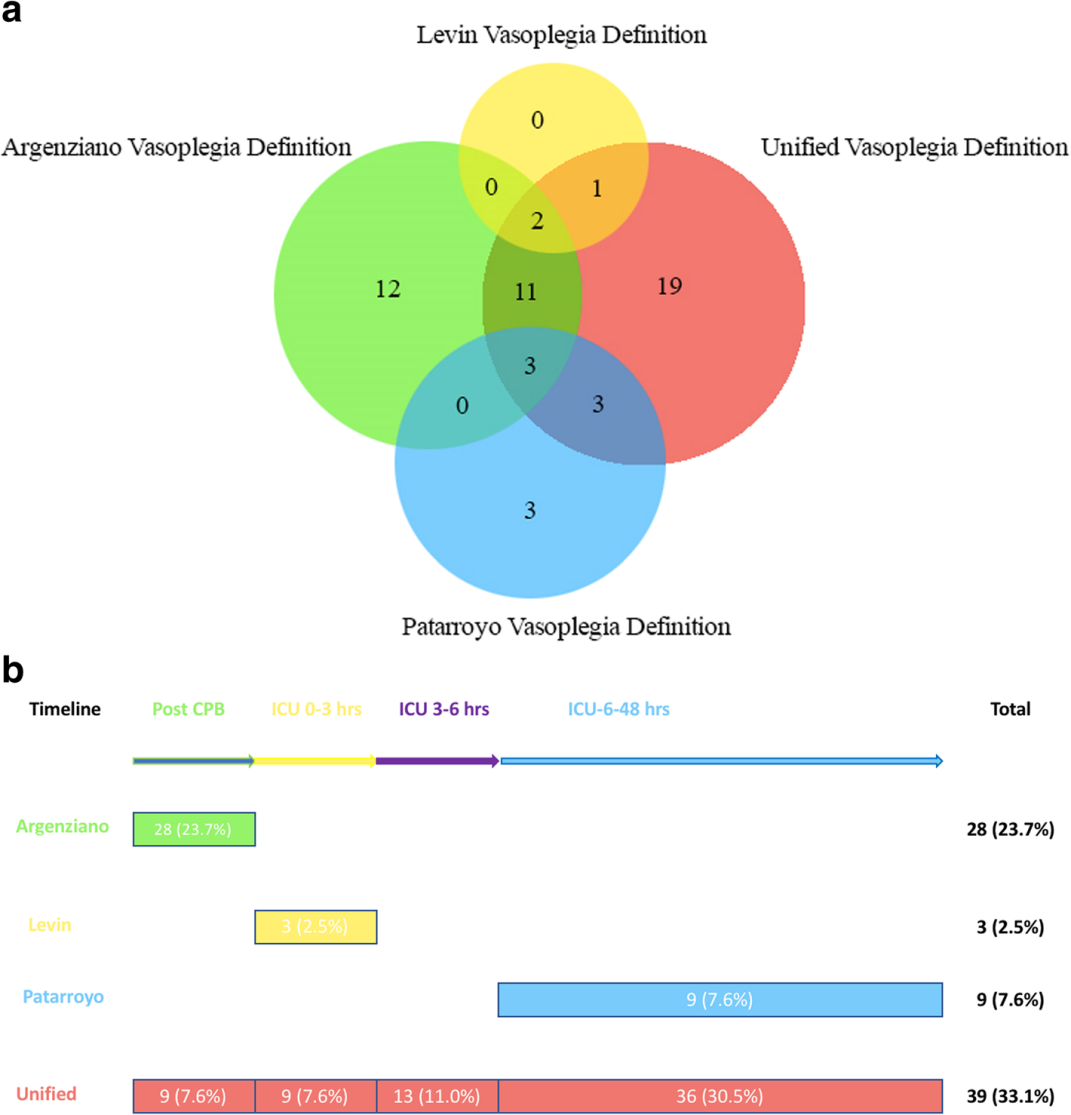
Stratification of vasoplegic patients according to the definition (Fig. 1a) and time frame of vasoplegia (Fig. 1b). Figure 1a. Overlap of the number of vasoplegic patients according to various definitions. Figure 1b. Time line of vasoplegia development in patients (numbers(%)) according to various definitions and time frames

The incidence of vasoplegia was 33.1% using the unified definition for the entire duration of our observation period. The Argenziano definition focusses on the early post bypass period and applying their criteria identified 28 patients (23.7%) as vasoplegic in our cohort and 9 patients (7.6%) using our unified definition (Fig. 1a, b). Employing the Levin criteria for the first 3 postoperative ICU hours revealed only 3 patients as vasoplegic (2.5%) (Fig. 1a) while applying our proposed unified criteria for the same time frame revealed a higher number of 9 patients (7.6%). Closer analysis revealed that the discrepancy was related to CVP criteria as postoperative CVP levels remained high (> 8 mmHg) in our patient cohort despite cfLVAD therapy. If the CVP criteria were omitted from the Levin definition, we would have identified 10 patients (8.5%) as vasoplegic in this time period. Applying the Patarroyo definition for the 6–48 postoperative hours revealed 9 patients (7.6%) as vasoplegic (Fig. 1a) while using our proposed unified definition identified 36 patients (30.5%) being vasoplegic.

Since the existing definitions cover different perioperative periods, it is important to investigate if they capture the same patients (Fig. 1a). Only 2 patients were present in both the Argenziano and Levin groups and only 3 patients were common between the Argenziano and Patarroyo groups. There was no overlap between the Levin and the Patarroyo group. Sixteen patients met both the Argenziano and our proposed unified criteria. Our unified definition also captured all vasoplegic individuals identified by the Levin definition and 6 out of 9 vasoplegic patients by the Patarroyo definition.

As the unified definition covers the entire postoperative period, it is interesting to investigate the onset and the duration of early vasoplegia and the potential of late onset vasoplegia. Such analysis suggests that most patients presenting with vasoplegia immediately after cardiopulmonary bypass (CPB) (9 patients), remained vasoplegic for the early postoperative period in the ICU (8 patients up to 24 h and 4 patients up to 48 h). Furthermore, the onset, time frame and duration of vasoplegia appears different in these patients allowing identification of potential subgroups with unique pathophysiological patterns.

In addition, subgroup analysis using the unified definition revealed an incidence of vasoplegia of 52.6% (10 patients) in the short-term cfLVAD (Centrimag) group (19 patients) and a 29.3% (29 patients) incidence of vasoplegia in the long-term cfLVAD (Heartware-Heartmate II) group (99 patients).

### Association of vasoplegia with clinical outcomes

**Fig. 2:**
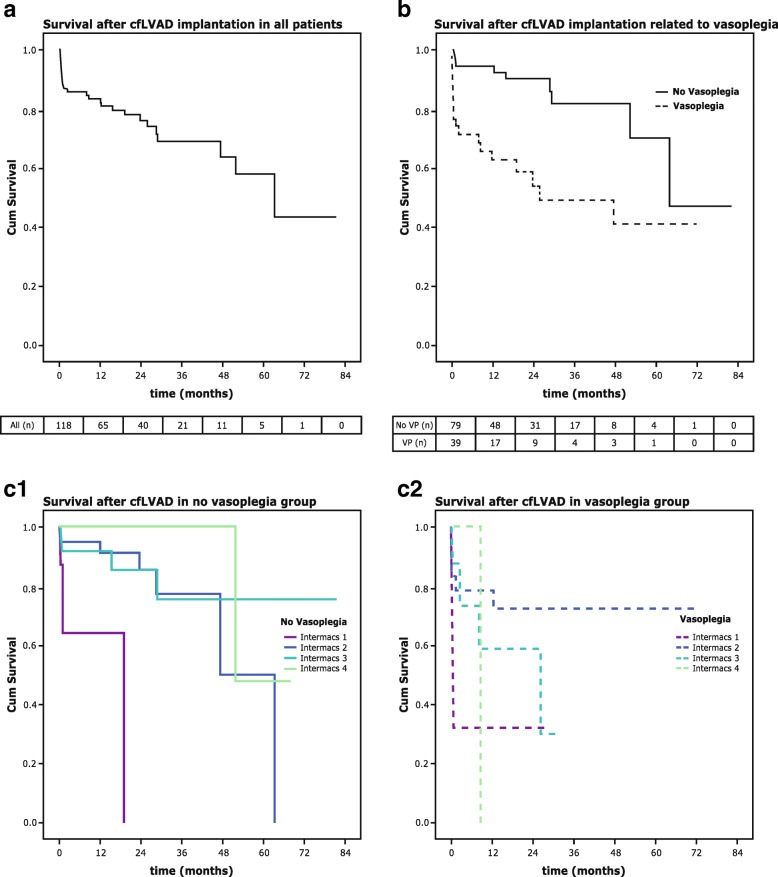
Patient survival after cfLVAD implantation in all patients in the derivation cohort (Fig. 2a), in patients with and without vasoplegia in the derivation cohort (Fig. 2b) censoring at heart transplantation or device removal (*p* <  0.01) and in patients with and without vasoplegia and stratified to Intermacs score (Fig. 2c)

Vasoplegia was associated with higher risks of postoperative adverse events, such as renal failure, stroke, bleeding and mortality, regardless of the definition used (Additional file 1: Table S1). For instance, patients with vasoplegia using the Argenziano definition developed renal failure more often (OR 3.2, 95% CI 1.3–8.0) and showed a higher VIS on the first postoperative day (OR 1.0, 95% CI 1.0–1.0). There were no significant differences in outcome using the Levin definition. Vasoplegic patients according to the Patarroyo definition developed renal failure more often (OR 4.4, 95% CI 1.1–17.8), had increased requirements for continuous veno-venous hemofiltration CVVH (OR 7.3, 95% CI 1.8–30.0), and developed more often a stroke (OR 11.7, 95% CI 2.5–53.3) and gastrointestinal bleeding (OR 5.0, 95% CI 1.1–22.9) during ICU stay. Moreover, the ICU mortality (OR 6.5, 95% CI 1.5–27.4) and 30-days mortality (OR 7.9, 95% CI 1.8–34.3) were higher in the vasoplegia group. Patients meeting the criteria of our unified definition of vasoplegia developed renal failure more often (OR 6.0, 95% CI 2.4–15.0), had increased requirements for CVVH (OR 7.9, 95% CI 2.6–23.6) and showed a higher VIS on the first and second postoperative day. They also required resternotomy for bleeding/tamponade more frequently in the first 48 postoperative hours (OR 3.0, 95%CI 1.2–7.8). Moreover, the ICU-LOS and the Post-ICU-Hospital-LOS were significantly longer. The crude ICU mortality (OR 5.8, 95%CI 1.9–18.2) and one-year mortality (OR 3.9, 95%CI 1.5–10.2) were higher in the vasoplegia group. Long-term patient survival after cfLVAD implantation censored at device removal or HTx was significantly worse in patients with vasoplegia compared to no vasoplegia patients (Log-Rank *p* <  0.01 (Fig. 2a and b) and remained statistically significant when patients were stratified according to their Intermacs score (*p* <  0.01) (Fig. 2c1 and Fig. 2c2).

### Prediction model

Univariable and multivariable logistic regression analysis of potential preoperative predictors for every definition of vasoplegia for the derivation cohort are reported in Table 3. Interestingly, only a few preoperative factors showed association with vasoplegia using the different existing vasoplegia definitions. Notably, we found no common preoperative predictors of vasoplegia using the older vasoplegia definitions, except previous cardiothoracic surgery and preoperative treatment with dopamine appearing as predictors of vasoplegia using the Patarroyo definition and our unified definition.

**Table 3:** Independent predictors of vasoplegia using previous definitions of vasoplegia and the unified definition

Argenziano				Univariable		Multivariable	
Preoperative characteristics	No vasoplegia *n* = 90	Vasoplegia *n* = 28	*P*-value	OR (95% CI)	P-value	OR (95% CI)	P-value
Pre-op dobutamine	43 (47.8%)	19 (67.9%)	0.04	2.65 (1.05–6.68)	0.04	2.65 (1.05–6.68)	0.04
Pre-op VIS	4.3 [0.0–7.3]	6.5 [4.0–11.9]	0.03	1.03 (1.00–1.06)	0.05		
Neutrophil/ Lymphocyt –ratio	4.5 [3.6–7.7]	8.0 [5.6–9.5]	0.04	1.13 (1.00 1.28)	0.05		
Levin				Univariable		Multivariable	
Preoperative characteristics	No vasoplegia *n* = 115	Vasoplegia *n* = 3	P-value	OR (95% CI)	*P*-value	OR (95% CI)	P-value
NCCMP	3 (2.6%)	2 (66.7%)	< 0.01	74.67 (5.22–1067.12)	< 0.01		1.00
DCMP	85 (73.9%)	0 (0.0%)	< 0.01	0.0 (0.0-∞)	1.00		
Clopidogrel	6 (5.2%)	1 (33.3%)	0.04	9.08 (0.71–114.92)	0.09		
Insuline	4 (3.5%)	1 (33.3%)	0.01	13.88 (1.03–186.60)	< 0.05		1.00
Preop norepinephrine	12 (10.4%)	3 (100%)	< 0.01	17.17 (1.45–203.67)	0.02		1.00
Lactate	1.9 [1.4–2.7]	13.1	0.03	∞ (0.00-∞)	0.98		
Patarroyo				Univariable	Multivariable		
Preoperative characteristics	No vasoplegia *n* = 109	Vasoplegia n = 9	P-value	OR (95% CI)	P-value	OR (95% CI)	P-value
Previous CT-surgery	10 (9.2%)	4 (44.4%)	< 0.01	7.92 (1.83–34.30)	< 0.01	10.4 (2.04–53.08)	< 0.01
Pre-op dopamine	22 (20.2%)	5 (55.5%)	0.02	4.94 (1.22–19.96)	0.03	6.46 (1.36–30.55)	0.02
Unified definition				Univariable		Multivariable	
Preoperative characteristics	No vasoplegia *n* = 79	Vasoplegia *n* = 39	P-value	OR (95% CI)	P-value	OR (95% CI)	P-value
Previous CT-surgery	5 (6.3%)	9 (23.1%)	< 0.01	4.44 (1.38–14.34)	0.01	7.60 (1.98–29.24)	< 0.01
Euroscore II*	20.6 [15.3–25.1]	32.6 [20.6–42.2]	< 0.01	1.06 (1.03–1.10)	< 0.01		
Pre-op dopamine	13 (16.5%)	14 (35.9%)	0.02	2.84 (1.17–6.88)	0.02	3.83 (1.28–11.46)	0.02
Pre-op VIS	4.1 [0.0–6.7]	6.5 [4.0–10.6]	< 0.01	1.04 (1.00–1.08)	0.06		
Bilirubin (μmol·l^−1^)	27.0 [15.5–37.3]	36.0 [25.0–44.5]	< 0.01	1.04 (1.01–1.07)	< 0.01	1.04 (1.01–2.08)	< 0.01
Creatinine* (μmol·l^−1^)	101 [91–150]	157 [117–186]	0.02	1.01 (1.01–1.02)	< 0.01		
Creatinine Clearance (ml·min^− 1^)	72 ± 29	57 ± 22	< 0.01	0.97 (0.95–0.99)	< 0.01	0.97 (0.95–0.99)	< 0.01

**Fig. 3:**
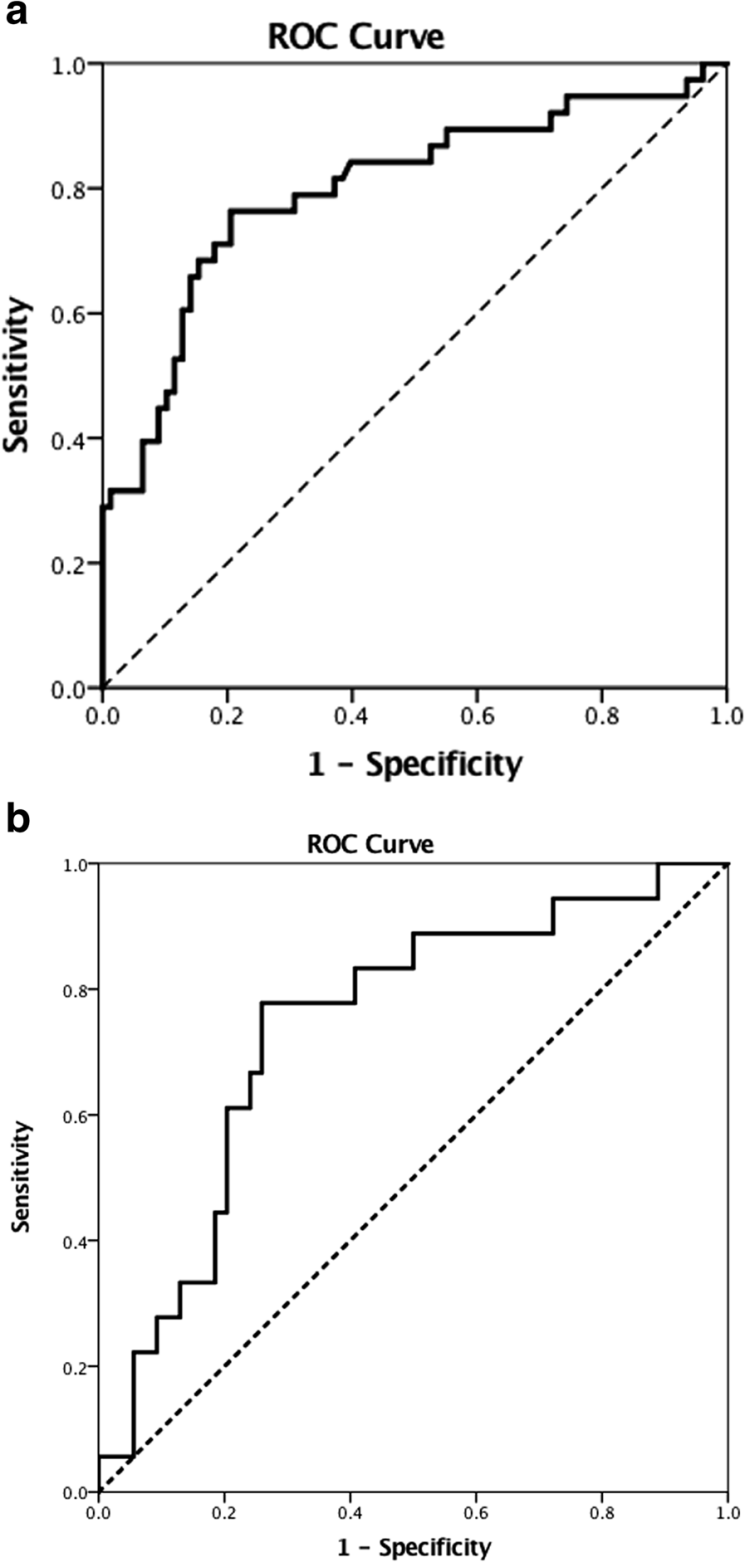
C-statistics of the final model and the temporal validation using the unified definition. Figure 3a. The predictive value of the final model, including previous cardiothoracic surgery, preoperative dopamine use, preoperative bilirubin level and creatinine clearance, calculated as area under the curve (AUC = 0.80, 95% CI 0.71–0.89, *P* <  0.01). Figure 3b. The predictive value of the temporal validation 0.74 (95% CI 0.61–0.87, *p* <  0.01)

Using our unified definition, previous cardiothoracic surgery, preoperative treatment with dopamine, higher bilirubin and creatinine levels, a lower creatinine clearance and a higher Euroscore II remained significantly associated with a higher risk of postoperative vasoplegia in the multivariable analysis (Additional file 2: Table S2, Additional file 2: Table S3). Due to multicollinearity, Euroscore II (a prediction model based on some of the other predictors [22]) and creatinine were excluded from the multivariable regression. The remaining 4 factors were independent predictors. The risk to develop vasoplegia after primary cfLVAD implantation can be calculated using the following formula: Predicted probability = e^(prediction score)^/(1+ e^(prediction score)^) and the prediction score as follows: − 0.542 + 1.88 * Previous cardiothoracic surgery + 1.383 * preoperative use of dopamine + 0.041 * preoperative total bilirubin − 0.032 * creatinine clearance. This final prediction model had a good discriminative ability (AUC = 0.80, 95%CI 0.71–0.89, *p* <  0.01) (Fig. 3a). Using the Youden index, the best cut-off point for predicting vasoplegia was 0.34 (sensitivity 76.3% and specificity 79.5%).

The temporal validation dataset consisted of 73 patients scheduled for cfLVAD implantation in our hospital in the period 2014–2016. Unfortunately, 1 patient died during the operation and data of 72 patients were available for further analysis. The incidence of vasoplegia in this validation cohort using the unified definition was 23.6% (17 patients). The AUC of our final prediction model in this validation cohort was 0.74 (95%CI 0.61–0.87, p <  0.01) (Fig. 3b).

### Subgroup analysis of long-term cfLVAD patients

The incidence of postoperative vasoplegia was 29.3% if we only consider the long-term assist device patients in the derivation cohort. Significant differences between the vasoplegia and non-vasoplegia group were BMI, previous cardiothoracic surgery, preoperative Euroscore II, LMWH, use of dopamine, preoperative VIS-score, lymphocytes, bilirubin levels and creatinine clearance. Univariable and multivariable logistic regression analysis of potential preoperative predictors of vasoplegia (unified definition) in these long-term cfLVAD group for the derivation cohort identified previous cardiothoracic surgery (OR 6.9, 95% CI 1.70–28.25), LMWH (OR 0.08, 95% CI 0.01–0.47) and preoperative use of dopamine (OR 6.21, 95% CI 6.21–20.96) as independent predictors (AUC 0.78, 95% CI 0.68–0.88).

### Intraoperative factors associated with postoperative vasoplegia

Regarding the intraoperative period, patients with vasoplegia using the unified definition were more likely to be treated with dopamine (38.5% vs. 19.0%, *p* = 0.02) (Additional file 3: Table S4). Furthermore, factors related to previous cardiothoracic surgery, such as skin-to-skin time, use of units fresh frozen plasma, units of blood platelets and cell saver blood were significantly different between patients developing vasoplegia and those who did not develop vasoplegia (Additional file 4: Table S4).

## Discussion

This study has comprehensively evaluated the most commonly used vasoplegia definitions and explored a new unified definition for the special conditions of cfLVAD implantation. Using the unified definition, vasoplegia remains a prevalent (33%) and clinically important condition, that was associated with important adverse clinical outcomes, such as renal failure, reinterventions, prolonged ICU-LOS, increased ICU mortality and a diminished survival over time. Previous cardiothoracic surgery, preoperative treatment with dopamine, preoperative bilirubin levels and preoperative creatinine clearance appear as independent predictors of postoperative vasoplegia with a good discriminative ability.

### Justification of proposed definition

To comprehensively address the issue of vasoplegia, we applied three previous definitions used in various fields of cardiac surgery [1, 2, 9]. We found that these definitions are based on different (cut-off values of) hemodynamic parameters at different time frames after surgery and consequently identified completely different patients as being vasoplegic. The definitions have major limitations in the setting of cfLVAD implantation. Argenziano’s definition used a liberal MAP threshold measured 5 min after weaning from CPB [1]. However, the immediate post-CPB period is characterized by rapid hemodynamic changes due to optimizing pump speed, changing ventricular geometry, adjusting inotropic support based on CO and RV function, and dynamic alterations in RV and LV preload following administration of fluids and/or blood products and protamine. The Levin’s criteria include the requirement for very low CVP < 5 mmHg. This is problematic in the end stage heart failure patients due to ventricular interdependence, biventricular failure, tricuspid regurgitation, and the clinical need of adequate RV preload to fulfil adequate preload of the cfLVAD. Specifically, our study shows that almost all cfLVAD patients exhibit higher CVPs at all stages during their ICU stay. Our conclusion therefore is that the Levin definition is not suitable for the special situation of cfLVAD implantation to define vasoplegia. Moreover, both Argenziano and Levin stratified patients using a single snap shot of clinical conditions excluding late onset vasoplegia in the ICU [1, 2]. In contrast, Patarroyo included less stringent hemodynamic values, more stringent vasoactive drug requirements, but excluded early onset vasoplegia [9].

For these crucial reasons, we developed and proposed a unified definition to capture vasoplegic patients in the full postoperative period after cfLVAD implantation. We aimed at capturing those patients who had very low MAP and/or low SVR (identical to Levin and Patarroyo [2, 9], but more stringent than Argenziano [1]) in the setting of normal cfLVAD flow, requiring higher vasoconstrictor treatment. On this point of vasoconstrictor treatment, our unified definition is more stringent than Argenziano and Levin, but less stringent than Patarroyo, who included at least two high dose vasoconstrictors.

### Incidence of vasoplegia after cfLVAD implantation

According to our unified definition, one-third of the patients were vasoplegic. Applying the Argenziano definition to our study allowed direct comparison of early post bypass vasoplegia between the first generation LVAD implantation (Argenziano study) and the new generation of cfLVAD surgery (our current study). In such comparison, it appears that the insertion of the newer generation of cfLVADs is associated with a lower incidence of vasoplegia (24%) compared to vasoplegia after pLVAD implantation (42%) [1]. Moreover, it is remarkable that the incidence of vasoplegia after cfLVAD insertion is higher compared to the incidence of vasoplegia after routine cardiac surgery [1–6] and HTx [8, 9].

### Vasoplegia and postoperative outcomes

While our study supports the recognition of improved outcomes after cfLVAD implantation compared to pLVADs [23, 24], it clearly demonstrates that vasoplegia and its sequelae are associated with postoperative outcomes representing an importantly increased risk for mortality. Moreover, nearly all ICU outcomes including renal failure, ICU stay, bleeding/tamponade were higher in vasoplegic patients (unified definition) compared to patients without vasoplegia. Thus, vasoplegia may be one of the most important determinants of the perioperative course and recovery in patients requiring mechanical circulatory support. The exact reasons and (molecular) mechanisms for such inferior outcomes of vasoplegia remain to be fully explored.

### Independent predictors of vasoplegia

By analysing predictive factors for vasoplegia in patients specifically scheduled for cfLVAD implantation, our study represents a unique approach on the field. Recently, van Vessem and coworkers published their predictive models for vasoplegia, but they included a heterogeneous group of 225 patients with only 14% of their patients being LVAD recipients [12]. Using our unified definition, we observed that the occurrence of postoperative vasoplegia was independently related to previous cardiothoracic surgery, preoperative treatment with dopamine, preoperative bilirubin levels and preoperative creatinine clearance.

In literature, there are conflicting data on the influence of previous cardiothoracic surgery on the development of vasoplegia [3, 7]. Our observations are in line with Patarroyo [9], who identified previous cardiothoracic surgery as an independent predictor of vasoplegia. This might be related to more complex and longer surgery, increased perioperative bleeding and transfusion requirements, longer skin-to-skin times, and inflammatory response. A primed inflammatory state and imbalance of vasoactive mediators may explain our observations with the associated intraoperative factors. We found that the preoperative administration of dopamine is an independent predictor of postoperative vasoplegia. We can only speculate about the reason for this phenomenon. We think it may relate to the status of heart failure in that these patients suffer more severe forms of heart failure requiring this mode of inotropic support. Such patients may suffer from vascular dysregulation and gut hypoperfusion having an impact on subsequent inflammatory response, cytokine elaboration and changes in mediators’ underlying vascular tone. Another plausible biological mechanism could be dopamine induced desensitisation of receptors involved in vasoconstriction such as down-regulation of beta and AT1 receptors [25] [26] causing catecholamine resistance. Increased bilirubin may indicate hepatic dysfunction associated with end-stage heart failure and altered hepatic degradation of circulating vasodilators [27–29]. Similarly, renal dysfunction, characterized by decreased preoperative creatinine clearance may also influence renal breakdown and elimination of various circulating vasodilators, such as bradykinin [30]. Further molecular studies are required to clarify the contribution of these alterations to vasoplegia.

We observed a somewhat lower predictive accuracy (lower AUC) in the temporal validation cohort compared to the derivation cohort. A prediction model typically performs less in a validation cohort, probably due to changed practice patterns over time. For instance, the incidence of vasoplegia in the validation cohort (23.6%) was somewhat less than the derivation cohort, we had more patients with previous cardiothoracic surgery (35.3% versus 23.1%) (Table 2) and less patients treated with dopamine in the validation cohort compared to the derivation cohort (5.9% versus 35.9%) (Supplemental material Table 2). Therefore, although this model is promising, the clinical utility of the predictive model remains unknown. Obviously, some factors cannot be changed but it may help in the decision pathway to tailor the inotropic agent of choice and the timing of the LVAD insertion with a better renal and liver function.

### Limitations

This is a retrospective study from a single institution with the usual limitations of such uncontrolled studies. Nevertheless, this design has allowed us to achieve our principle aim to highlight the incidence of vasoplegia and the impact on short-term outcome and survival. While we have undertaken a rigorous study and applied all major previous definitions of vasoplegia to the LVAD setting, we have realised these all have major shortcomings for the current application and needed to redefine vasoplegia for this setting. We believe that our concept unifies previous definitions and certainly identifies a very high-risk population, the unified definition needs to be agreed by wider international consensus and validated by prospective multicentre studies. In addition, the current study does not consider the perioperative metabolic state or inflammatory profiles of our cfLVAD patients [30–32]. These important aspects will be investigated in the near future. Moreover, the study did not focus on specific types of cfLVAD. This is an interesting area but such investigation will require cooperation from different centres and will be subject of future plans. We also have limitations due to the sample size, as we were restricted to include only a limited number of variables in the multivariable analysis to predict postoperative vasoplegia [31]. Inotropic scores, such as the Vasoactive Inotropic Score should be used with caution, because several vasoactive drugs result in vasoconstriction and/or in vasodilation, depending on the used doses and depending on the combination of used vasoactive agents. Moreover, the relative strength of action on the vasomotor tone of these vasoactive drugs compared to others is not fully understood. Finally, our focus was on prediction of vasoplegia using preoperative independent predictors. However, the incidence of vasoplegia might be influenced by associated intraoperative factors, such as intraoperative transfusions [32], the use of CBP and the duration of CPB [3], as reported previously.

## Conclusion

Previous definitions of vasoplegia all have limitations in their applicability to patients after cfLVAD implantation. Using our unified definition, vasoplegia affects about one-third of the patients after cfLVAD implantation. Despite successful surgery and cfLVAD performance, affected patients suffer more often from serious postoperative complications, such as prolonged ICU and hospital stay, increased renal failure, and markedly reduced survival. We identified previous cardiothoracic surgery, preoperative treatment with dopamine, preoperative bilirubin levels and preoperative creatinine clearance as independent preoperative predictors.

## Additional files

Additional file 1: **Table S1.** Postoperative data of patients in the derivation cohort for every vasoplegia definition. Values are expressed as numbers (and %), or median [Interquartile range]. CVVH = continuous veno-venous hemofiltration, Hb = Hemoglobin, ICU = intensive care unit, RV = right ventricular, RVAD = right ventricular assist device, VIS = vasoactive inotropic score. (DOCX 22 kb)

Additional file 2: **Table S2.** Preoperative medication in patients in the derivation and validation cohort. Values are expressed as numbers and % of patients, mean ± SD, or median [Interquartile Range]. ACE-inhibitors = Angiotensin Converting Enzyme inhibitors; ARB = Angiotensin Receptor Blocker; IS = Inotropic Score; LMWH = Low Molecular Weight Heparin; VIS = Vasoactive Inotropic Score. (DOCX 18 kb)

Additional file 3: **Table S3.** Pre-op laboratory data in patients in the derivation and validation cohort. Values are expressed as mean ± SD or median [Interquartile Range]. ALAT = Alanine Amino-Transferase, ASAT = Aspartate Amino-Transferase, BNP = Brain Natriuretic Peptide, CRP = C-reactive protein, Hb = Haemoglobin, RDW = Red Cell Distribution Width. (DOCX 16 kb)

Additional file 4: **Table S4.** Intraoperative data in patients in the derivation cohort. Values are expressed as numbers and % of patients, mean ± SD, or median [Interquartile Range]. CS = Cell Saver, FFP = Fresh Frozen Plasma, HR = Heart Rate, PC = Packed Cells. (DOCX 15 kb)

## Abbreviations

cfLVAD: Continuous flow left ventricular assist device
CI: Cardiac index
CPB: Cardiopulmonary bypass
CVP: Central venous pressure
CVVH: Continuous veno-venous hemofiltration
EZIS: Electronic hospital information system
HMIIRS: Heartmate II risk score
HTx: Heart transplantation
ICU: Intensive care unit
LOS: Length of stay
LVAD: Left ventricular assist device
MAP: Mean arterial pressure
OR: Odds ratio
pLVAD: Pulsatile left ventricular assist device
RV: Right ventricle
SVR: Systemic vascular resistance
VIS: Vaso-active inotropic score
