# Supplementary material for: Vasoplegia after implantation of a continuous flow left ventricular assist device: incidence, outcomes and predictors
Source: BMC Anesthesiol. 2018 Dec 8;18:185. doi: 10.1186/s12871-018-0645-y (PMC6286572; doi:10.1186/s12871-018-0645-y)
Supplement: Supplementary file 1 — Table S1. Postoperative data of patients in the derivation cohort for every vasoplegia definition. Values are expressed as numbers (and %), or median [Interquartile range]. CVVH = continuous veno-venous hemofiltration, Hb = Hemoglobin, ICU = intensive care unit, RV = right ventricular, RVAD = right ventricular assist device, VIS = vasoactive inotropic score. (DOCX 22 kb) [file 12871_2018_645_MOESM1_ESM.docx]

**Supplemental material table 1.**

| Postoperative data | Argenziano | | | Levin | | | Patarroyo | | | Unified definition | | |
| --- | --- | --- | --- | --- | --- | --- | --- | --- | --- | --- | --- | --- |
|  | No vasoplegia n = 90 | Vasoplegia n = 28 | P-value | No vasoplegia n = 115 | Vasoplegia n = 3 | P-value | No vasoplegia n = 109 | Vasoplegia n = 9 | P-value | No vasoplegia n = 79 | Vasoplegia n = 39 | P-value |
| First Hb after ICU-arrival (g·dl^-1^) | 10.1 [8.4-11.2] | 8.5 [7.9-9.9] | 0.007 | 9.7 [8.2-11.0] | 9.3 [8.7-9.7] | 0.747 | 9.7 [8.2-11.0] | 8.7 [9.2-10.1] | 0.356 | 10.1 [8.5-11.3] | 8.5 [7.9-10.1] | 0.001 |
| **Inotropic scores** | | | | | | | | | | | | |
| VIS max first 24 hours | 33.5 [18.7-60.0] | 54.0 [32.2-84.0] | 0.001 | 37.4 [20.8-65.0] | 61.3 [50.8-74.3] | 0.218 | 37.4 [21.1-64.3] | 60.1 [25.5-87.8] | 0.131 | 27.6 [17.6-44.0] | 67.5 [51.1-85.6] | 0.000 |
| VIS max second 24 hours | 20.0 [9.1-43.5] | 26.9 [14.2-58.8] | 0.096 | 21.8 [9.7-44.7] | 45.9 [42.4-50.5] | 0.109 | 21.8 [9.2-43.8] | 59.8 [20.0-73.0] | 0.018 | 14.5 [6.7-25.7] | 50.0 [34.6-61.5] | 0.000 |
| **Outcome data** | | | | | | | | | | | | |
| Resternotomy during ICU stay | 30 (33.3%) | 9 (32.1%) | 0.907 | 38 (33.0%) | 1 (33.3%) | 0.992 | 35 (32.1%) | 4 (4.4%) | 0.450 | 17 (21.5%) | 22 (56.4%) | 0.000 |
| Resternotomy first 48 hours due to bleeding/tamponade | 16 (17.8%) | 6 (21.4%) | 0.665 | 22 (19.1%) | 0 (0.0%) | 0.401 | 20 (18.3%) | 2 (2.2%) | 0.774 | 8 (10.1%) | 14 (35.9%) | 0.001 |
| Open chest treatment | 3 (3.3%) | 4 (14.3%) | 0.032 | 7 (6.1%) | 0 (0.0%) | 0.660 | 7 (6.4%) | 0 (0.0%) | 0.433 | 3 (3.8%) | 4 (10.3%) | 0.162 |
| Mediastinitis | 1 (1.1%) | 0 (0.0%) | 0.575 | 1 (0.9%) | 0 (0.0%) | 0.871 | 1 (0.9%) | 0 (0.0%) | 0.773 | 1 (1.3%) | 0 (0.0%) | 0.480 |
| Renal failure | 17 (18.9%) | 12 (42.9%) | 0.010 | 28 (24.3%) | 1 (33.3%) | 0.721 | 24 (22.0%) | 5 (5.6%) | 0.025 | 12 (15.2%) | 17 (43.6%) | 0.001 |
| CVVH | 13 (14.4%) | 8 (28.6%) | 0.088 | 20 (17.4%) | 1 (33.3%) | 0.476 | 16 (14.7%) | 5 (5.6%) | 0.002 | 7 (8.9%) | 14 (35.9%) | 0.000 |
| Stroke | 8 (8.9%) | 3 (10.7%) | 0.772 | 10 (8.7%) | 1 (33.3%) | 0.147 | 7 (6.4%) | 4 (44.4%) | 0.000 | 6 (7.6%) | 5 (12.8%) | 0.358 |
| RV-failure | 50 (55.6%) | 14 (50.0%) | 0.606 | 63 (54.8%) | 1 (33.3%) | 0.462 | 59 (54.1%) | 5 (5.6%) | 0.934 | 41 (51.9%) | 20 (51.3%) | 0.651 |
| RVAD implant after ICU-admission | 4 (4.4%) | 0 (0.0%) | 0.256 | 4 (3.5%) | 0 (0.0%) | 0.742 | 4 (3.7%) | 0 (0.0%) | 0.559 | 1 (1.3%) | 3 (7.7%) | 0.070 |
| Gastrointestinal bleeding | 10 (11.1%) | 3 (10.7%) | 0.953 | 12 (10.4%) | 1 (33.3%) | 0.211 | 10 (9.2%) | 3 (3.3%) | 0.026 | 6 (7.6%) | 7 (17.9%) | 0.091 |
| Pneumonia | 12 (13.3%) | 2 (7.1%) | 0.376 | 13 (11.3%) | 1 (33.3%) | 0.244 | 13 (11.9%) | 1 (1.1%) | 0.942 | 10 (12.7%) | 4 (10.3%) | 0.704 |
| Pump thrombosis | 12 (13.3%) | 1 (3.6%) | 0.150 | 13 (11.3%) | 0 (0.0%) | 0.537 | 12 (11.0%) | 1 (1.1%) | 0.993 | 9 11.4%) | 4 (10.3%) | 0.853 |
| Lactate acidosis | 3 (3.3%) | 2 (7.1%) | 0.382 | 5 (4.3%) | 0 (0.0%) | 0.712 | 4 (3.7%) | 1 (1.1%) | 0.287 | 2 (2.5%) | 3 (7.7%) | 0.191 |
| Delirium | 10 (11.1%) | 5 (17.9%) | 0.349 | 15 (13.0%) | 0 (0.0%) | 0.503 | 14 (12.8%) | 1 (1.1%) | 0.881 | 10 (12.7%) | 5 (12.8%) | 0.980 |
| ICU mortality | 11 (12.2%) | 5 (17.9%) | 0.447 | 15 (13.0%) | 1 (33.3%) | 0.311 | 12 (11.0%) | 4 (4.4%) | 0.005 | 5 (6.3%) | 11 (28.2%) | 0.001 |
| ICU-Length-of-stay (days) | 7.0 [5.0-14.7] | 6.8 [4.8-12.1] | 0.889 | 6.9 [4.9-12.9] | 40.5 [6.0-74.9] | 0.355 | 7.0 [4.9-12.7] | 6.9 [5.4-51.4] | 0.403 | 6.1 [4.6-10.4] | 10.5 [6.9-20.8] | 0.002 |
| Post-ICU-Hospital-Length-of-stay (days) | 24.5 [16.5-33.5] | 24.9 [19.7-34.8] | 0.432 | 24.5 [17.4-33.5] | 37.0 [26.5-47.5] | 0.278 | 24.5 [17.4-33.4] | 31.5 [15.5-61.0] | 0.434 | 23.4 [17.0-31.0] | 28.5 [21.2-47.5] | 0.024 |
| 30-days mortality | 9 (10.0%) | 5 (17.9%) | 0.261 | 13 (11.3%) | 1 (33.3%) | 0.244 | 10 (9.2%) | 4 (4.4%) | 0.002 | 6 (7.6%) | 8 (20.5%) | 0.041 |
| 1-year mortality | 14 (15.6%) | 8 (28.6%) | 0.122 | 21 (18.3%) | 1 (33.3%) | 0.508 | 18 (16.5%) | 4 (4.4%) | 0.039 | 9 (11.4%) | 13 (33.3%) | 0.004 |
