# Supplementary material for: Vasoplegia after implantation of a continuous flow left ventricular assist device: incidence, outcomes and predictors
Source: BMC Anesthesiol. 2018 Dec 8;18:185. doi: 10.1186/s12871-018-0645-y (PMC6286572; doi:10.1186/s12871-018-0645-y)
Supplement: Supplementary file 2 — Table S2. Preoperative medication in patients in the derivation and validation cohort. Values are expressed as numbers and % of patients, mean ± SD, or median [Interquartile Range]. ACE-inhibitors = Angiotensin Converting Enzyme inhibitors; ARB = Angiotensin Receptor Blocker; IS = Inotropic Score; LMWH = Low Molecular Weight Heparin; VIS = Vasoactive Inotropic Score. (DOCX 18 kb) [file 12871_2018_645_MOESM2_ESM.docx]

**Supplemental material table 2.** Preoperative medication in patients in the derivation and validation cohort.

| Preoperative medication | **Derivation cohort** | | | **Validation cohort** | | |
| --- | --- | --- | --- | --- | --- | --- |
| Unified vasoplegia definition | No vasoplegia n = 79 | Vasoplegia  n = 39 | P-value | No vasoplegia n = 55 | Vasoplegia  n = 17 | P-value |
| Aspirin | 11 (13.9%) | 2 (5.1%) | 0.15 | 3 (5.5%) | 3 (17.6%) | 0.11 |
| Coumadin | 52 (65.8%) | 27 (69.2%) | 0.71 | 37 (67.3%) | 13 (69.2%) | 0.47 |
| LMWH | 23 (29.1%) | 5 (12.8%) | 0.05 | 9 (16.4%) | 1 (5.9%) | 0.28 |
| Heparin | 1 (1.3%) | 0 (0.0%) | 0.48 | 1 (1.8%) | 0 (0.0%) | 0.58 |
| Clopidogrel | 5 (6.3%) | 2 (5.1%) | 0.80 | 1 (1.8%) | 1 (5.9%) | 0.37 |
| Dabigatran | 1 (1.3%) | 0 (0.0%) | 0.48 | 0 (0.0%) | 0 (0.0%) |  |
| Beta-blockers | 25 (31.6%) | 9 (23.1%) | 0.33 | 23 (41.8%) | 6 (35.3%) | 0.63 |
| ACE-inhibitors | 32 (40.5%) | 18 (46.2%) | 0.56 | 35 (63.6%) | 4 (23.5%) | <0.01 |
| ARB | 9 (11.4%) | 4 (10.3%) | 0.85 | 7 (12.7%) | 3 (17.6%) | 0.61 |
| Ca-entry Blockers | 0 (0.0%) | 1 (2.6%) | 0.39 | 1 (1.8%) | 0 (0.0%) | 0.58 |
| Amiodarone | 28 (35.4%) | 17 (43.6%) | 0.09 | 15 (27.3%) | 9 (52.9%) | 0.05 |
| Kinidine | 1 (1.3%) | 0 (0.0%) | 0.48 | 1 (1.8%) | 0 (0.0%) | 0.58 |
| Digoxin | 6 (7.6%) | 4 10.3%) | 0.63 | 7 (12.7%) | 5 (29.4%) | 0.11 |
| Pronestyl | 1 (1.3%) | 0 (0.0%) | 0.48 | 0 (0.0%) | 0 (0.0%) |  |
| Loop-diuretics | 64 (81.0%) | 36 (92.3%) | 0.11 | 53 (96.4%) | 15 (88.2%) | 0.20 |
| Thiazide-diuretics | 5 (6.3%) | 3 (9.1%) | 0.78 | 2 (3.6%) | 2 (11.8%) | 0.20 |
| K-sparing diuretics | 54 (68.4%) | 31 (39.2%) | 0.21 | 47 (85.5%) | 15 (88.2%) | 0.77 |
| Nitrates | 5 (6.7%) | 3 (7.7%) | 0.78 | 6 (10.9%) | 1 (5.9%) | 0.54 |
| Oral antidiabetics | 2 (2.7%) | 2 (5.1%) | 0.46 | 6 (10.9%) | 2 (11.8%) | 0.92 |
| Insulin | 3 (3.8%) | 2 (5.1%) | 0.74 | 4 (7.3%) | 0 (0.0%) | 0.25 |
| Gastric protection | 46 (58.2%) | 16 (41.0%) | 0.08 GROUPS=New_DefdeWaal(0 1)  /MISSING=ANALYSIS  /VARIABLES= gewicht lengte BSADubois  /CRITERIA=CI(.95). | 21 (38.2%) | 10 (58.8%) | 0.13 GROUPS=New_DefdeWaal(0 1)  /MISSING=ANALYSIS  /VARIABLES= gewicht lengte BSADubois  /CRITERIA=CI(.95). |
| Thyroid medication | 7 (8.8%) | 0 (0.0%) | 0.06 | 5 (9.1%) | 1 (5.9%) | 0.68 |
| Milrinone | 34 (43.0%) | 20 (51.3%) | 0.40 | 20 (36.4%) | 8 (47.1%) | 0.43 |
| Dobutamine | 40 (50.6%) | 22 (56.4%) | 0.55 | 29 (52.7%) | 10 (58.8%) | 0.71 |
| Norepinephrine | 7 (8.8%) | 7 (17.9%) | 0.15 | 0 (0.0%) | 0 (0.0%) |  |
| Dopamine | 13 (16.5%) | 14 (35.9%) | 0.02 | 4 (7.3%) | 1 (5.9%) | 0.84 |
| Nitroglycerin IV | 1 (1.3%) | 0 (0.0%) | 0.48 | 1 (1.8%) | 0 (0.0%) | 0.58 |
| IS | 2.4 ± 2.5 | 3.4 ± 3.1 | 0.06 | 1.7 ± 1.9  /TABLES= thiazidediuretica Ksparenddiureticum Nitraten0nee1ja OAD insuline1 Maagbeschermers0nee1ja BY New_DefdeWaal  /FORMAT=AVALUE TABLES  /STATISTICS=CHISQ  /CELLS=COUNT  /COUNT ROUND CELL. ± 2.5 | 2.3 ± 2.1 | 0.33 |
| VIS | 4.1 [0.0-6.7] | 6.5 [4.0-10.6] | <0.01 | 2.8 [0.0-4.5] | 4.1 [0.0-6.2] | 0.20 |
