# Supplementary material for: Vasoplegia after implantation of a continuous flow left ventricular assist device: incidence, outcomes and predictors
Source: BMC Anesthesiol. 2018 Dec 8;18:185. doi: 10.1186/s12871-018-0645-y (PMC6286572; doi:10.1186/s12871-018-0645-y)
Supplement: Supplementary file 3 — Table S3. Pre-op laboratory data in patients in the derivation and validation cohort. Values are expressed as mean ± SD or median [Interquartile Range]. ALAT = Alanine Amino-Transferase, ASAT = Aspartate Amino-Transferase, BNP = Brain Natriuretic Peptide, CRP = C-reactive protein, Hb = Haemoglobin, RDW = Red Cell Distribution Width. (DOCX 16 kb) [file 12871_2018_645_MOESM3_ESM.docx]

**Supplemental material table 3.** Pre-op laboratory data in patients in the derivation and validation cohort.

| **Pre-op laboratory data** | **Derivation cohort** | | | **Validation cohort** | | |
| --- | --- | --- | --- | --- | --- | --- |
| Unified vasoplegia definition | No vasoplegia n = 79 | Vasoplegia n = 39 | P-value | No vasoplegia n = 55 | Vasoplegia n = 17 | P-value |
| Leucocytes (*10^9^·l^-1^) | 12.2 [8.4-14.9] | 10.4 [8.8-12.2] | 0.41 | 8.6 [7.5-10.9] | 7.7 [6.6-8.6] | 0.10 |
| Neutrophils (*10^9^·l^-1^) | 10.0 [6.3-11.3] | 8.2 [6.5-9.0] | 0.46 | 6.0 [4.6-7.8] | 5.0 [4.4-6.3] | 0.27 |
| Lymphocytes (*10^9^·l^-1^) | 1.7 [1.4-2.4] | 1.2 [0.6-1.6] | 0.08 | 1.7 [1.1-2.1] | 1.1 [0.8-1.5] | 0.02 |
| Neutrophil/Lymphocyt ratio | 4.5 [3.4-7.8] | 6.7 [4.4-18.8] | 0.19 | 3.5 [2.6-5.8] | 4.7 [3.4-8.1] | 0.10 |
| Volume neutrophils (AU) | 148 [142-155] | 149 [144-161] | 0.99 | 139 [132-150] | 133 [130-145] | 0.26 |
| CRP (mg·l^-1^) | 56 [27-109] | 64 [41-90] | 0.15 | 6 [4-27] | 15 [7-36] | 0.14 |
| Hb (g·dl^-1^) | 12.2 ± 1.8 | 12.3 ± 1.8 | 0.74 | 13.3 ± 1.7 | 11.8 ± 1.8 | <0.01 |
| RDW (%CV) | 14.1 [13.5-14.6] | 15.1 [13.2-15.9] | 0.09 | 14.2 [12.8-15.9] | 15.0 [14.3-17.6] | 0.02 |
| Platelets (*10^9^·l^-1^) | 226 [161-307] | 189 [141-274] | 0.16 | 199 [166-255] | 218 [149-256] | 0.66 |
| ASAT (U·l^-1^) | 70 [43-551] | 60 [36-742] | 0.67 | 38 [28-52] | 38 [26-68] | 0.96 |
| ALAT (U·l^-1^) | 179 [43-720] | 163 [41-1277] | 0.46 | 56 [26-115] | 27 [20-61] | 0.16 |
| Bilirubin total (μmol·l^-1^) | 27.0 [15.5-37.3] | 36.0 [25.0-44.5] | <0.01 | 26.5 [15.8-46.3] | 28.0 [23.0-40.0] | 0.52 |
| Creatinine (μmol·l^-1^) | 101 [91-150] | 157 [117-186] | 0.02 | 101 [80-129] | 147 [121-266] | <0.01 |
| Creatinine clearance (ml·min^-1^) | 72 ± 29 | 57 ± 22 | <0.01 | 79 ± 341 | 56 ± 27 | <0.01 |
| BNP (pmol·l^-1^) | 650 [350-812] | 1172 [466-1464] | 0.10 | 378 [213-632] | 625 [319-853] | 0.20 |
| Lactate (mmol·l^-1^) | 1.9 [1.3-2.4] | 1.9 [1.5-2.1] | 0.12 | 1.7 [1.4-2.3] | 1.7 [1.4-2.4] | 0.87 |
