# Supplementary material for: Vasoplegia after implantation of a continuous flow left ventricular assist device: incidence, outcomes and predictors
Source: BMC Anesthesiol. 2018 Dec 8;18:185. doi: 10.1186/s12871-018-0645-y (PMC6286572; doi:10.1186/s12871-018-0645-y)
Supplement: Supplementary file 4 — Table S4. Intraoperative data in patients in the derivation cohort. Values are expressed as numbers and % of patients, mean ± SD, or median [Interquartile Range]. CS = Cell Saver, FFP = Fresh Frozen Plasma, HR = Heart Rate, PC = Packed Cells. (DOCX 15 kb) [file 12871_2018_645_MOESM4_ESM.docx]

**Supplemental material table 4**

Intraoperative data in patients in the derivation cohort.

| **Intraoperative data** | | | |
| --- | --- | --- | --- |
| Unified vasoplegia definition | No vasoplegia | Vasoplegia | P-value |
|  | n = 79 | n = 39 |  |
| First HR (bpm) | 100 [80-118] | 100 [90-120] | 0.47 |
| First rectal temp (**°**C**)** | 37.1 ± 0.8 | 37.0 ± 1.0 | 0.58 |
| Skin-to-skin time (min) | 212 [185-253] | 233 [191-355] | 0.04 |
| CPB-time (min) | 95 [80-115] | 107 [83-123] | 0.16 |
| Ao-clamp (n) | 4 (5.1%) | 4 (10.3%) |  |
| Ao-clamp time (min) | 34 ± 26 | 40 ± 24 | 0.74 |
| **Intraoperative medication** | | | |
| Dexamethason | 44 (55.7%) | 23 (59.0%) | 0.74 |
| Milrinone | 75 (94.9%) | 38 (97.4%) | 0.53 |
| Dobutamine | 70 (88.6%) | 36 (92.3%) | 0.53 |
| Norepinephrine | 77 (97.5%) | 37 (94.9%) | 0.46 |
| Levosimendan | 0 (0.0%) | 1 (2.6%) | 0.15 |
| Dopamine | 15 (19.0%) | 15 (38.5%) | 0.02 |
| Epinephrine | 6 (7.6%) | 4 (10.3%) | 0.63 |
| Nitric Oxide | 65 (82.3%) | 39 (100.0%) | 0.50 |
| **Intraoperative transfusion** | | | |
| PC (units) | 0 [0-2] | 0 [0-2] | 0.45 |
| FFP (units) | 1 [0-3] | 4 [2-5] | <0.01 |
| Platelets (units) | 0 [0-1] | 1 [0-2] | <0.01 |
| CS-blood (ml) | 0 [0-500] | 500 [0-1000] | 0.02 |
| Autologous Blood (ml) | 0 [0-1000] | 0 [0-500] | 0.39 |
| **LVAD implanted** | | | |
| CM | 9 (11.4%) | 10 (25.6%) | 0.14 |
| HMII | 63 (79.7%) | 26 (66.7%) |  |
| HW | 7 (8.8%) | 3 (7.7%) |  |
| HMIII | 0 | 0 |  |
